# Mechanical ventilation in medical departments: a necessary evil, or a blessing in bad disguise?

**DOI:** 10.1186/s13584-019-0322-8

**Published:** 2019-06-03

**Authors:** Yuval Schwartz, Amir Jarjoui, Amos M. Yinnon

**Affiliations:** 1 Infectious Disease Unit, Shaare Zedek Medical Center, affiliated with the Hebrew University-Hadassah Medical School, Jerusalem, Israel; 2 Lung Institute, Shaare Zedek Medical Center, affiliated with the Hebrew University-Hadassah Medical School, Jerusalem, Israel; 3 Division of Internal Medicine, Shaare Zedek Medical Center, affiliated with the Hebrew University-Hadassah Medical School, P.O. Box 3235, 91031 Jerusalem, Israel

**Keywords:** Mechanical ventilation, Medical departments, End-of-life care, Intensive care unit, Advance directives, Terminal Extubation

## Abstract

In most countries there is a mismatch between demand for intensive care unit (ICU) beds and ICU bed availability. Because of a policy of low ICU-bed reimbursement this mismatch is much more profound in Israel, which arguably has the lowest number of ICU beds/1000 population of OECD countries. Increasing demand for mechanical ventilation has led to an ever-rising presence of ventilated patients in medical departments, which may reach up to 15% or more of medical beds, especially during winter months, posing serious challenges such as: delivery of adequate treatment, guaranteeing patient safety, nosocomial infections, emergence and spread of resistant organisms, dissatisfaction among family members and medical and nursing staff, as well as enormous direct and indirect expenses.

This paper assumes that no change in ICU reimbursement will occur in the near future. We, therefore, describe a number of policy issues that should ideally be addressed together in order to cope realistically with the increase in mechanically ventilated patients in medical departments. First, all medical departments should operate a 5-bed augmented care room with one dedicated nurse per shift. Medical residents should receive a mandatory 3-month ICU rotation in their first year of residency, and attending physicians should receive adequate training in mechanical ventilation and vasopressor support, point-of-care ultrasound and central venous catheterization. Second, family physicians should be required to discuss and fill relevant forms with advance directives for elderly and/or chronically ill patients. Third, rules for terminal extubation should be established, even if only applied infrequently. Finally, co-payment should be considered for families of patients demanding all possible medical treatment in spite of contrary medical advice, considering these patients’ terminal status.

Implementation of these recommendations will require policy decision making in the Ministry of Health, Scientific Council of the Israeli Medical Association, the professional societies (for internal medicine and family practice) and finally by the leadership of individual hospitals.

## Introduction

In Israeli hospitals, the Ministry of Health (MOH) policy of inadequate reimbursement for Intensive Care Unit (ICU) admissions has led to a paucity of ICU beds, and the rate of these per 1000 hospital beds is among the lowest in OECD countries [1, 2] On the other hand, cultural and religious attitudes consistently lead to families to request “to do everything possible” for their patients, including intubation and mechanical ventilation, even for chronically and terminally ill patients for whom the medical staff would not contemplate or recommend such intervention. The result has been a dramatic increase in critically ill patients, some of whom are mechanically ventilated, who are treated in regular medical wards, which are not staffed and equipped to provide optimal care for such patients.

In the article by Zisk-Rony et al. [3], which retrospectively analyzed all mechanically ventilated patients over 20 years in two hospitals in Jerusalem, Israel, it was clearly demonstrated that the overall ventilator-days on the medical wards increased over these two decades (from 4 patients/day in 1997 to 24 patients/day in 2016). This trend will probably continue in the next years, partially driven by the aging of the population and the use of new treatment modalities for chronic diseases. In addition, we know from previous research, also from this country, that ventilated patients in medical wards only have a 25–30% chance of survival, and many survivors remain chronically debilitated [4–7]. The authors are to be commended for providing meticulously collected and analyzed data on this issue and for raising it for professional and public debate. In our 1000-bed hospital too there are routinely 1.5–2.5 ventilated patients in medical departments for each patient in the 14-bed ICU, for a total of 25–30 patients or 10–15% of all internal medicine beds.

The Israel Medical Association should definitely submit to the MOH a demand for an increased percentage of ICU beds out of all hospital beds. However, in this country almost all medical departments are obliged to house multiple patients in hallways on a daily basis because of underfunding for these departments. An increase in the country’s medical budget will probably first lead to an increase in general medical bed availability. Therefore, this paper assumes that no change in ICU reimbursement will occur in the near future. We will discuss several practical as well as controversial solutions to this serious clinical, financial and ethical challenge facing the Israeli medical system and society at large (Table 1).

**Table 1:** The significant increase, and expected continued increase, in mechanically ventilated patients in medical departments. Policy recommendations

No.	Policy consideration	Current situation	Aim
1	An augmented care unit should be present in all departments of medicine.	90% of hospitals report presence, not necessary in all departments “(https://www.ima.org.il/mainsite/ScientificCouncil.aspx)”.	All medical departments.
2	Residents in internal medicine should have a 3-month mandatory rotation in the ICU, preferable in their first year.	Minority, almost none in their first year.	All residents, preferably as part of their medical (rather than elective) program.
3	Attending (senior) physicians should receive appropriate training in mechanical ventilation, vaso-pressor support, point-of-care US and central	Probably none.	National workshops, possibly with a medical simulations course + mandatory US course.
4	Advance directives regarding end-of-life care, intubation and resuscitation, to be deposited in an accessible central data bank.	Very, very few.	Either by amendment of the law or as Ministry of Health directive: family physicians should be required to discuss and fill relevant forms for elderly and/or chronically ill patients.
5	Terminal extubation	None	For selected cases, setting an appropriate mechanism.
6	Co-payment by families demanding “every possible treatment” contrary to medical advice.	So far families have the prerogative to demand tremendously costly medical care – even if medical advice is otherwise.	Co-payment should be a policy consideration if families demand mechanical ventilation in spite of medical advice.

### Augmented care rooms

First, most Israeli hospitals have responded to the rise of critically ill patients, included ventilated patients, for whom an ICU bed is unavailable, by setting up an augmented care room in medical departments [8]. However, many medical department directors report an absence of such a unit, and many more report dissatisfaction with its capabilities. These units, carrying various names including monitoring units, usually consist of 5 beds, electronic monitoring equipment and one nurse per shift, which is about twice the usual rate in the general medical ward. Patient rooms in medical departments usually house three patients; monitoring units conveniently consist of two combined rooms, with one bed sacrified to accommodate a small nursing station. These units’ staffing and equipment are significantly inferior to that of ICUs [8, 9]; e.g., medical care is provided by the internists staffing the departments. The Scientific Council of the Israeli Medical Association actually demands the presence of such a unit for each medical department as a prerequisite for accreditation “(https://www.ima.org.il/mainsite/ScientificCouncil.aspx)”. Several studies have demonstrated which critically ill patients would benefit from care in these augmented care units [10–12]. As has been pointed out by Zisk-Rony et al. [3], the absence of a dedicated intensivist physician mandates that all medical residents attend a 3-month rotation in ICU in order to obtain the minimally required expertise. Although medical residents recognize this need, many are inclined to choose rotations in other subspecialty units. Therefore, the Scientific Council should consider mandating a 3-month ICU rotation as part of the medical residency program. In addition, all attending internists staffing medical departments with augmented care rooms should undergo relevant training regarding invasive ventilation, intensive hemodynamic support, point-of-care ultrasound (US), US-guided endovascular cannulation, etc.

### End-of-life care and advance directives

Second, Zisk-Rony et al. [3] briefly mentioned the sensitive issue of end-of-life care. Physicians are obliged by law to inquire about the end-of-life wishes of all patients with a less than 6-month life expectancy. All internists taking care of critically ill patients in medical departments engage in discussions with family members regarding their critically ill relatives, of whom the vast majority did not issue advanced directives. In many instances there are differences of opinion among family members, especially siblings, and social pressure as well as religious attitudes often lead to a request “to do everything”, including intubation and mechanical ventilation. As a result, many elderly, chronically and critically ill patients are intubated, in face of medical advice to refrain from such care as it is deemed futile, causing unnecessary suffering and expenses. Requiring co-payment by families in these instances is probably both rational and a necessity, but quite likely socially unacceptable.

Community-based physicians in Israel do not initiate talks as a matter of routine with all their elderly and/or ill patients about end-of-life issues. We could draw inspiration from the American “conversation project” as well as from Atul Gawande’s book Being Mortal “(https://theconversationproject.org/)”, [13] about the importance of having these conversations and the important role that physicians could play in encouraging them to occur. Chronically ill and/or elderly patients should be advised to make a living-will that stipulates their wishes to be resuscitated (or not) and/or to be ventilated (or not). The 2005 law on terminally ill patients should be amended, or at least the Israeli Ministry of Health should issue a requirement for family physicians, to address this issue with their patients – while these are still relatively stable and ambulatory. This will ease the emotional burden on the family and hospital-based teams to decide whether to intubate an acute on chronically ill patient. This can save a lot of suffering and agony for the patient himself and their family and will decrease the expense of care in long-term care facilities (if the family is fortunate enough to find room in such facility).

### Terminal extubation

All of the above is possibly not sufficient or attainable in the near future. Therefore, it seems that Zisk-Rony et al. [3] have set the stage for discussion of an even more sensitive issue, terminal extubation (TE). Although we refer to “terminal extubation” in this commentary, a more appropriate, but less commonly used term, has been advocated: “compassionate extubation” [14–16].

Physicians are acutely familiar with the principle “primum non nocere” (above all, do no harm), but we occasionally fail to recall that the oath ascribed to Hippocrates includes a commitment not to over-treat patients who are “overmastered by their disease”. Terminal extubation indicates the withdrawal of mechanical ventilation from patients who are not expected to regain independent respiration. In these patients, mechanical ventilation is prolonging the patient’s dying process and removing it at the patient’s or their surrogate’s request, allows nature to follow its course.

Only in 1914 after the famous case of *Schloendorff v. Society of New York Hospital*, 105 N.E. 92 (N.Y. 1914), the principle of “respondeat superior” was established in US law. In this case, Mary Schloendorff from San Francisco was admitted to New York Hospital to evaluate a stomach disorder. A fibroid tumor was diagnosed and surgical removal recommended, which Schloendorff adamantly refused. She consented to an examination under anesthesia, during which the doctors removed the tumor. Afterwards, Schloendorff developed gangrene of the left arm. Schloendorff sued, and won. Justice Benjamin Cardozo wrote in the Court’s opinion: “Every human being of adult years and sound mind has a right to determine what shall be done with his own body; and a surgeon who performs an operation without his patient’s consent commits an assault for which he is liable in damages. This is true except in cases of emergency where the patient is unconscious and where it is necessary to operate before consent can be obtained” [17–19].

The notion that this principle also applies to life-sustaining treatment emerged only in 1976, in the case of *Karen Ann Quinlan*. This young woman lost consciousness and stopped breathing after a party. Medical intervention saved her life, but a lack of oxygen left her in a persistent, vegetative state. After several months without change in her condition, Karen’s parents requested the removal of their daughter’s ventilator. The hospital, together with the Quinlans, commenced a legal battle against the Morris County, New Jersey, prosecutor over whether the withdrawal of life support constituted homicide. On March 31, 1976, in a landmark decision, the New Jersey State Supreme Court ruled (7–0) and acknowledged the right of a patient to refuse even life-sustaining treatment and that right still stands even if the patient loses capacity. The ruling also emphasized the importance of the local ethics’ committees in these conflicts [20, 21].

Another important case, relevant to the issue of mechanical ventilation in medical departments raised by Zisk-Rony et al. [3], was brought up before the California District Court of Appeal in 1983. In the *Barber v. Superior Court* a patient called Herbert suffered cardiac arrest and went into coma. Two physicians determined that Herbert had sustained significant brain damage and the chance of recovering was deemed extremely small. This information was conveyed to Herbert’s family and upon their request, the respirator was removed. The two involved physicians were charged with murder but were eventually acquitted [22]. This case also demonstrates the right of families, recognized by US law, to make decisions for comatose patients even if they are not their court-appointed custodians.

These cases and others, combined with the ability of medical treatment to prolong life but not necessary to improve the quality of life, has led to an increased interest (in both the medical and ethics literature) about futile medicine. The Annals of Internal Medicine has published a set of criteria for “medical futility” [23]. According to the latter article “Although exceptions and caution should be borne in mind, we submit that physicians can judge a treatment to be futile and are entitled to withhold a procedure on this basis”. Although professional medical and ethics’ societies have failed to reach an agreement about the definition and exact criteria for “medical futility”, TE has been widely practiced around the world, and many physicians believe that TE is medically, morally and ethically justified to shortcut protracted suffering, that can only be expected to lead to death [24, 25]. However, in Israel, aside from the discussed obstacles to TE, there is a profound religious aspect, which will probably constitute the ultimate barrier against adoption of TE, perhaps allowing it only for very rare and publicly acknowledged cases. There is a profound fear for the “slippery slope” phenomenon that could lead to ever more serious immoral practices, with which the general and medical history is replete [26–30].

### In balance

One of us has witnessed terminal extubation during a fellowship in the US in the early 1990s and vividly remembers the associated emotional and moral shock, also expressed by others [24] that doctors should save lives, relieve suffering, and never be in a situation to shortcut life. Having said this, the only logical conclusion we can draw as physicians and a society, is that the law should be amended, or at least the Ministry of Health (MOH) should issue an explicit obligation, to induce family physicians to discuss end-of-life requests and draw up and sign official documents for elderly and/or chronically ill patients to be submitted and saved by the central depository set up for this purpose by the MOH. This is expected to reduce the number of such patients who receive mechanical ventilation, receive sub-optimal care in medical departments and subsequently die after much suffering. A discussion of the financial implications for society at large of mechanical ventilation of large numbers of terminally ill patients is beyond the scope of this commentary, but should also be considered by policy makers.

### In conclusion

The policy changes suggested in this paper could lead to a change from a “necessary evil” situation to one that could be considered a “blessing in disguise”. Rather than refraining from intubation if there is no ICU bed available – as practiced in many western countries – or from being overly compliant with family demands “to do everything” – as currently happens in Israeli hospitals - we would enter a new era in which mechanical ventilation in elderly, chronically ill patients is limited to patients with a reasonable chance for recovery, who can expect to receive better care than is currently available in medical departments.

## Data Availability

Not applicable.

## Abbreviations

ICU: Intensive care unit
MOH: Ministry of Health
OECD: Organisation for Economic Co-operation and Development
TE: Terminal extubation
US: Ultrasound
